# Comparing executive functions in children with attention deficit hyperactivity disorder with or without reading disability: A resting‐state EEG study

**DOI:** 10.1002/brb3.2951

**Published:** 2023-03-07

**Authors:** Maryam Tabiee, Ahmad Azhdarloo, Mohammad Azhdarloo

**Affiliations:** ^1^ Department of Foreign Languages and Linguistics, School of Literature and Humanities Shiraz University Shiraz Iran; ^2^ Department of Psychology Islamic Azad University of Arsanjan Branch Arsanjan Fars Iran; ^3^ Department of Psychology Islamic Azad University of Marvdasht Branch Marvdasht Fars Iran

**Keywords:** ADHD, coherence, executive functions, reading disability, resting‐state EEG

## Abstract

**Introduction:**

As numerous studies have shown, executive dysfunction is the main impairment in attention‐deficit/hyperactivity disorder. According to recent neuroimaging studies, the frontoparietal coherence plays a key role in overall cognitive functions. Therefore, the aim of this study was to compare executive functions during resting‐state EEG by monitoring brain connectivity (coherence) patterns in children with attention deficit hyperactivity disorder (ADHD) with or without reading disability (RD).

**Methods:**

The statistical sample of the study consisted of 32 children with ADHD aged between 8 and 12 years old with or without specific RD. Each group consisted of 11 boys and 5 girls that were matched on chronological age and gender. EEG was recorded during eyes‐opened condition and brain connectivity within and between frontal and parietal regions was analyzed within theta, alpha, and beta bands.

**Results:**

The results revealed that across the frontal regions, the comorbid group showed a significant reduction in the left intrahemispheric coherence in the alpha and beta bands. The ADHD‐alone group exhibited increased theta and decreased alpha and beta coherence in frontal regions. In the frontoparietal regions, children in the comorbid group showed lower coherence between frontal and parietal networks compared to children without comorbid RD.

**Conclusion:**

The findings indicate that brain connectivity (coherence) patterns of children with ADHD with comorbid RD were more abnormal and lend support to more disrupted cortical connectivity in the comorbid group. Thus, these findings can be a useful marker for better recognizing ADHD and comorbid disabilities.

## Graphical abstract

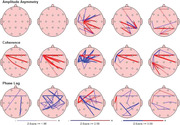

## 1 INTRODUCTION

Attention‐deficit hyperactivity disorder (ADHD) is known as one of the major neurodevelopmental conditions in children that estimated to affect 3%–5% of this group (APA, 2013; Asherson, 2012). According to recent extensive studies, the major characteristics in individuals with ADHD consist of impairments in internal control of attention, planning, inhibition, verbal, and spatial working memory (Biederman et al., 2008; Kasper et al., 2012; Kofler et al., 2011). A consistent body of evidence has associated ADHD with anomalies of cognitive functions that according to Barkley's characterization, these main symptoms of ADHD group underlie executive dysfunction. Therefore, impairment in executive function is a core cause of ADHD (Goldstein & Naglieri, 2008).

It seems that individuals with ADHD experience comorbid disorders. Among these conditions, specific reading disability (RD) is one of the most frequent comorbid ones, occurring approximately in 25%–40% of individuals with ADHD (DuPaul et al., 2013). Similarly, specific RD is reported to be associated with impairments in executive functions. Studies have reported that individuals with specific reading disabilities experienced significant problems in executive functions, including verbal and visuospatial working memory, inhibition, and organization that are mostly performed by the prefrontal and parietal lobe (Akyurek, 2018; Booth et al., 2010).

Executive functions are a set of interrelated cognitive abilities that allow individuals to control and coordinate other cognitive activities like memory, language, and visuospatial ability (Diamond, 2013; Nowrangi et al., 2014). Some examples of executive functioning identified include planning, paying attention, working memory, problem solving, and inhibitory control (Akyurek, 2018; Moura et al., 2015). Executive functions play a significant role in learning. One of the most important hypotheses regarding the causes of ADHD is that executive dysfunctions in individuals with ADHD usually continue until adulthood and lead to learning disorders that have negative effects on the social, behavioral, and academic skills of this group. In particular, executive dysfunctions have a negative impact on learning (Dvorsky & Langberg, 2019).

A growing body of neuroimaging studies has shown that executive dysfunctions in individuals with ADHD are associated with abnormalities in prefrontal cortex (Moore et al., 2012). It has been shown that in addition to frontal regions, parietal cortical regions, cerebellar lobes, and basal ganglia are also closely linked to executive functions (Nowrangi et al., 2014). fMRI studies revealed that areas related to executive control containing prefrontal cortex, inferior frontal junction, and parietal regions (van Son et al., 2019). It is due to this connectivity component that the frontoparietal coherence plays a key role in overall cognitive functions (Shaw et al., 2015). In accord with this idea, numerous studies, including fMRI, ERP, and EEG, have highlighted that the most common cognitive deficits in individuals with ADHD are related to frontal, basal ganglia, or parietal cortical dysfunction (Hoogman et al., 2017; Norman et al., 2016; Rubia et al., 2016). Thus, ADHD is associated with atypical brain functional connectivity (Barry et al., 2002), and these abnormal resting EEGs could be a good predictor of cognitive impairment in ADHD (Emond et al., 2009; Schutter et al., 2006).

Halle et al. (2014) recently found that individuals with ADHD showed rightward beta asymmetry in all parietal indices which supports the deficiency of attention‐directed information processing and cognitive sets in this group. Lee et al. (2021) showed that elevated theta activity in the frontal region and reduced alpha activity in the posterior area were positively associated with aberrant inhibitory control in the ADHD group. Similarly, some EEG studies have reported atypical brain functional connectivity in individuals with reading disabilities (Arns et al., 2007; Shiota et al., 2000). For example, Rippon and Brunswick (2000) investigated the EEG changes in tasks that reported reading difficulties. They found elevated right parietal–occipital activation in beta activity and high frontal theta activity during phonological task in dyslexic children. In this regard, Rippon and Brunswick suggested that both alpha synchronization and elevated theta activity can be marker of poor attentional and memory processes in dyslexics.

In the last few years, most practitioners of neuropsychological characteristics strongly favor the use of electroencephalography to investigate the relationship between these characteristics and brain activity. In fact, electroencephalography contains information about the structure of neural networks, and its analysis can greatly help us acquiring information about abnormal brain and cognitive functions, brain arousal (e.g., relative power, absolute power, and peak frequency), and connective (coherence, phase) characters. With this explanation, the recording of electrical waves in the resting state (RS) provides the possibility to obtain information about how neural connections work without performing any cognitive tasks. Most importantly, according to Lord and Opaka‐Jeffrey (2016), examining the coherence index in the resting state contains important physiological information that is a reflection of how the brain integrates and processes information in the resting state and on the other hand, coordinates the activity of brain neurons.

In general, EEG coherence alludes to the functional connection between brain areas (Thornton & Carmody, 2017) that can provide significant information about brain activation and neuropsychological characteristics. It is well known that in addition to prefrontal cortical activation, patterns of frontoparietal coherence have also a crucial role in executive functions, and theta and beta oscillations are most relevant to this network (see Sauseng et al. 2006). As shown by previous EEG work, decreased theta and beta coherence between the frontal and parietal regions in the left hemisphere reflects poor executive function in patients with Parkinson's disease (Teramoto et al., 2016). Other evidence reveals that delta band connectivity in left parietal and central areas correlated with executive functions (Bong et al., 2020). Friedman et al. (2006) also stated that there is a tight link between the Alpha band and executive functions.

To date, few studies exist that have investigated EEG resting state related to executive functions. Basharpoor et al. (2021) examined EEG coherence in frontal regions in adults in both eyes‐closed and eyes‐opened conditions and showed that executive functions are marked by hypercoherence of the alpha, beta, and theta bands between left and right frontal regions, and beta and theta hypocoherence between frontal regions of the two hemispheres. Teramoto et al. (2016) examined the EEG coherence measures in Parkinson's disease in an eyes‐closed condition and showed that executive dysfunction in Parkinson's disease is associated with leftward hypocoherence between the frontal and parietal cortices in this group.

In contrast to the accumulative study related to the relationship between EEG bands and task‐based executive functioning, fewer studies explored the relationship between executive function and RS‐EEG coherence in children and adults. Among these limited studies that do exist, Zhang et al. (2018) examined EEG localized activation in executive function performance in children with ADHD. They noticed the reduced spectral power in the posterior region during EEG resting state in children with ADHD. The relation between alpha and beta bands in EEG resting state and task‐switching task was examined by Ambrosini and Vallesi (2016a). They found that there was a strong relationship between left beta/alpha activity in the middle frontal gyrus and transient cognitive control, and a strong relationship between right beta/alpha activity in the middle frontal gyrus and sustained cognitive control. In another study, Ambrosini and Vallesi (2016b) found that there is a strong relationship between left prefrontal cortex and inhibition in EEG RS‐state. These results can confirm the importance of fast waves (alpha/beta) in executive functions.

Although a good deal of research in the field of cognitive impairments in individuals with ADHD has investigated the main symptoms of executive dysfunction in this group, there is still insufficient evidence about the link between functional brain connectivity and executive function in the ADHD group and comorbid disorders. The aforementioned reviewed literature led to the hypotheses that the comorbid group compared to the ADHD group would have lower coherences within and between frontal and parietal regions in some frequency bands. Based on these considerations, this research project sets out to investigate the difference EEG variables related to executive dysfunctions in children with ADHD with comorbid RD and ADHD without RD. By examining EFs in RS‐EEG, this study can create more appropriate estimations and treatment for children with ADHD. Consistent with other EEG work, we specifically focused on theta (4–8 Hz), alpha (8–12 Hz), and beta (12–30 Hz) bands that are tightly linked to executive functions.

## 2 PARTICIPANTS

Thirty‐two native Iranian children aged between 8 and 12 years old participated in this study. A group of 16 children with ADHD with comorbid RD (11 boys and 5 girls; mean age = 9.1, SD = 1.25) and a group of 16 ADHD children without RD (11 boys and 5 girls; mean age = 9.3, SD = 1.41) were selected. In both groups, the children were matched on chronological age and gender. Both groups were recruited for participation through rehabilitation centers. The diagnoses for ADHD children in both groups were based on Persian version of the Wechsler Intelligence Scale for Children (WISC‐IV), the revised Canners’ parent rating scale, and clinical interviews check list. To observe ethical consideration, the parents were informed in writing about the goals and importance of the research and signed the informed consent form to enter the research. In addition, the participants could opt out of the study at any time. Parents were informed that all information about the subjects would remain confidential, and the names of the individuals would be avoided in the results.

### 2.1 Data recording

To perform this study, the participant sat in front of the laptop monitor and after cleaning the skin of the ears and forehead with medical alcohol, the special cap of the device was placed on his/her head. Based on the international 10–20 system, 19 electrodes for standard electroencephalography recording (including Fz, F7, F8, F3, F4, Cz, C3, C4, Fp1, Fp2, T3, T4, T5, T6, Pz, P3, P4, O1, and O2) were placed on the participant's skull. Electrodes A1 and A2 were placed on the ears as potential references. Electroencephalography recording was done by eWave amplifier. EEG recording was done during eyes opened resting state for 5 min, and during this time, the participants did not perform any tasks. Therefore, EEG recording was conducted in anterior (Fp1, Fp2, Fz, F7, F8, F3, F4), central (Cz, C3, C4, T3, T4), and posterior (T5, T6, Pz, P3, P4, O1, O2) areas. According to the prior arrangements, the children were requested not to take any medicine before the electroencephalography recording. All EEG recordings were done between certain hours of the day, that is, around 10–12 in the morning and after a normal night's sleep and breakfast, to make the recordings conditions the same and to avoid the influence of time. During recording, participants were asked to keep looking at the screen and minimize their movements and even not blink. The impedances for all electrodes were maintained below 5 k Ω.

### 2.2 Data analysis

After artifact correction and preprocessing, 30 epochs of 2 s with minimal artifacts were selected; totally for the final segments of EEG, we had 60 s to analyze. To extract quantitative data for each participant, the recorded EEG was analyzed offline using NeuroGuide software. Fast Fourier transform analysis was used to assess EEG coherence activity in all frequency bands. Therefore, a comparison was made between children with ADHD with comorbid RD and ADHD children without comorbid RD concerning the resting‐state EEG coherence in the theta, alpha, and beta bands for the following analysis: the means of coherence in left (FP1‐F3, FP1‐F7, and F3‐F7) and right (FP2‐F4, FP2‐F8, and F4‐F8) frontal intrahemispheric regions, means of coherence in frontal interhemispheric (FP1‐FP2, F3‐F4, and F7‐F8) regions and left (F3‐P3) and right (F4‐P4) frontoparietal regions. Statistical analyses were performed by SPSS software (version 23.0), and the significance level for all analyses was set at *p* < .05.

The analysis was considered according to two fixed‐effects factors; group (ADHD + RD children vs. ADHD children), and EEG coherence of theta, alpha and, beta bands. Before using the parametric analysis of variance test, Levene's test was used to meet its assumptions. As can be seen in Tables 1 and 2, based on the findings of the study, the assumption of equality of variances has been confirmed and the significance levels of Levene's test allow the use of parametric tests for all EEG frequency bands. Therefore, to explore within‐groups differences in EEG coherence in frontal and parietal regions, one‐way ANOVA test was used. To analyze between‐groups differences in EEG coherence in frontal and parietal regions in each frequency band, a *t*‐test was undertaken.

**TABLE 1:** *F* Levene's test for equality of variance of EEG coherences in the attention deficit hyperactivity disorder (ADHD)‐alone group.

*p* Value	*F*	EEG frequency bands
.578	.316	Theta
.522	.420	Alpha
.858	.33	Beta

**TABLE 2:** *F* Levene's test for equality of variance of EEG coherences in the ADHD + RD group

*p* Value	*F*	EEG frequency bands
.758	.096	Theta
.993	.000	Alpha
.715	.136	Beta

## 3 EEG WITHIN‐GROUP RESULTS

The ANOVA results for the EEG coherence analysis in frontal regions revealed significant group differences in three frontal regions in all three (theta, alpha, and beta) bands. As the results showed, the comorbid group exhibited significant EEG coherence changes across left and right frontal intrahemispheric, frontal interhemispheric, and frontoparietal regions in theta (*F* (6.44); df (4); *p* < .001), alpha (*F* (8.51); df (4); *p* < .001), and beta (*F* (4.45); df (4); *p* < .002) frequency bands. Similarly, children in the ADHD‐alone group showed significantly different coherences across all frontal interhemispheric and intrahemispheric regions and frontoparietal regions in theta (*F* (13.68); df (4); *p* < .001), alpha (*F* (17.90); df (4); *p* < .001), and beta (*F* (12.26); df (4); *p* < .001) bands. The results for the analysis of the frontal regions revealed that children in the ADHD‐alone group had significantly lower interhemispheric coherence in beta compared with left (*t* = 15.61, *p* < .001) and right (*t* = 12.36, *p* < .001) frontal intrahemispheric regions. In addition, the ADHD group without comorbid RD exhibited elevated coherence in the left frontal intrahemispheric in theta band (*t* = 17.75, *p* < .001) when this was compared to frontal interhemispheric region.

The results for EEG coherence in frontoparietal regions revealed that children with ADHD with comorbid RD had significantly lower theta (*t* = 16.61, *p* = . 007) and alpha (*t* = 18.36, *p* < .001) EEG coherence in the left frontoparietal region when this was compared to left intrahemispheric frontal region. The ADHD group without comorbid RD exhibited lower alpha coherence in the left frontoparietal region (*t* = 15.16, *p* = .015) compared to the left frontoparietal region.

## 4 EEG BETWEEN‐GROUP RESULTS

Totally, the results for EEG coherence across frontal regions revealed that the comorbid group had decreased coherence in the left frontal intrahemispheric of theta (*t* = 13.80, *p* = .024), alpha (*t* = 12.81, *p* = .050), and beta bands (*t* = 14.21, *p* = .017) when this was compared to left frontal intrahemispheric regions in the ADHD‐alone group.

**FIGURE 1:**
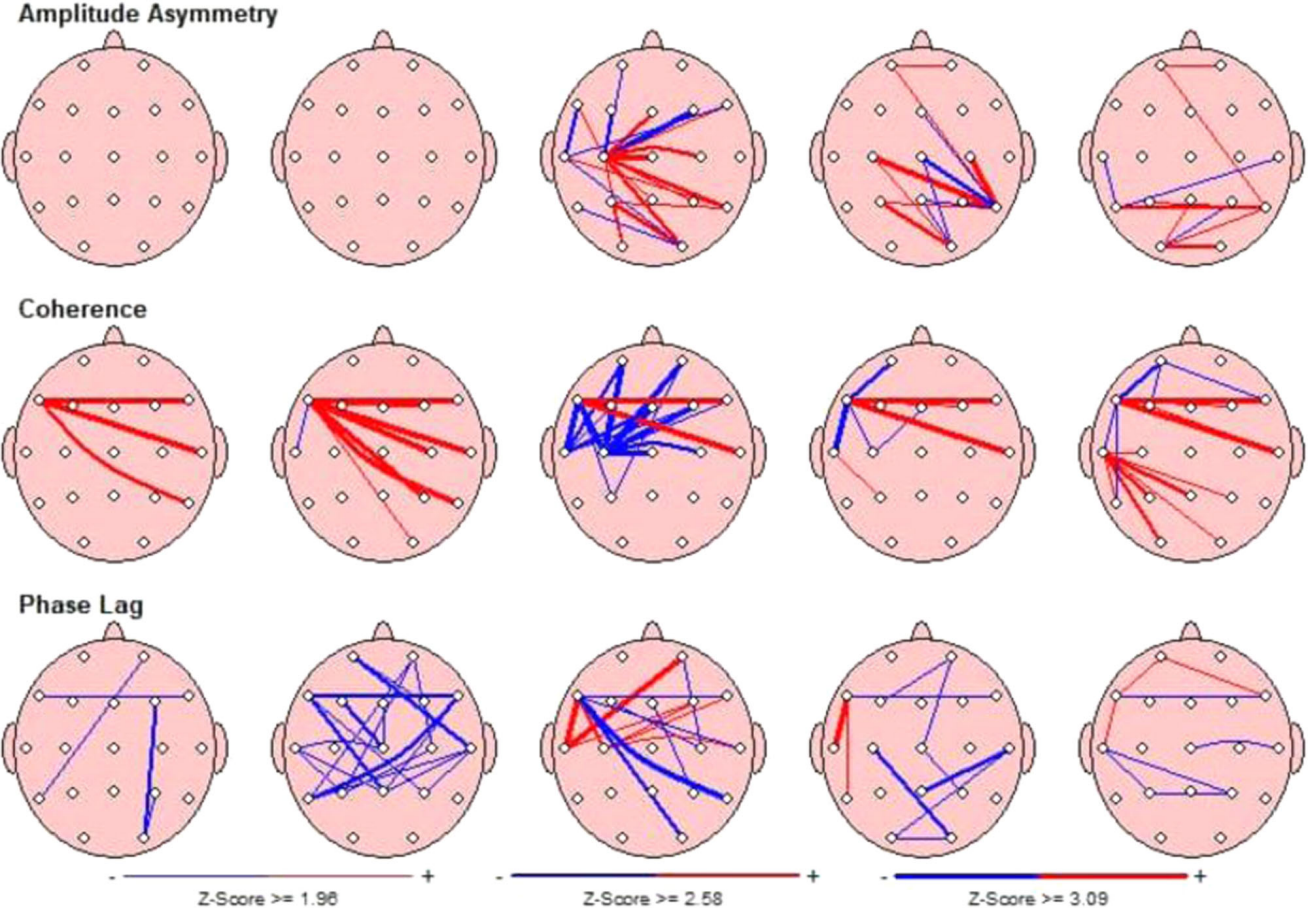
An example of QEEG summary maps in the attention deficit hyperactivity disorder (ADHD)‐alone group. Hypercoherence in theta band and hypocoherence in alpha band.

**FIGURE 2:**
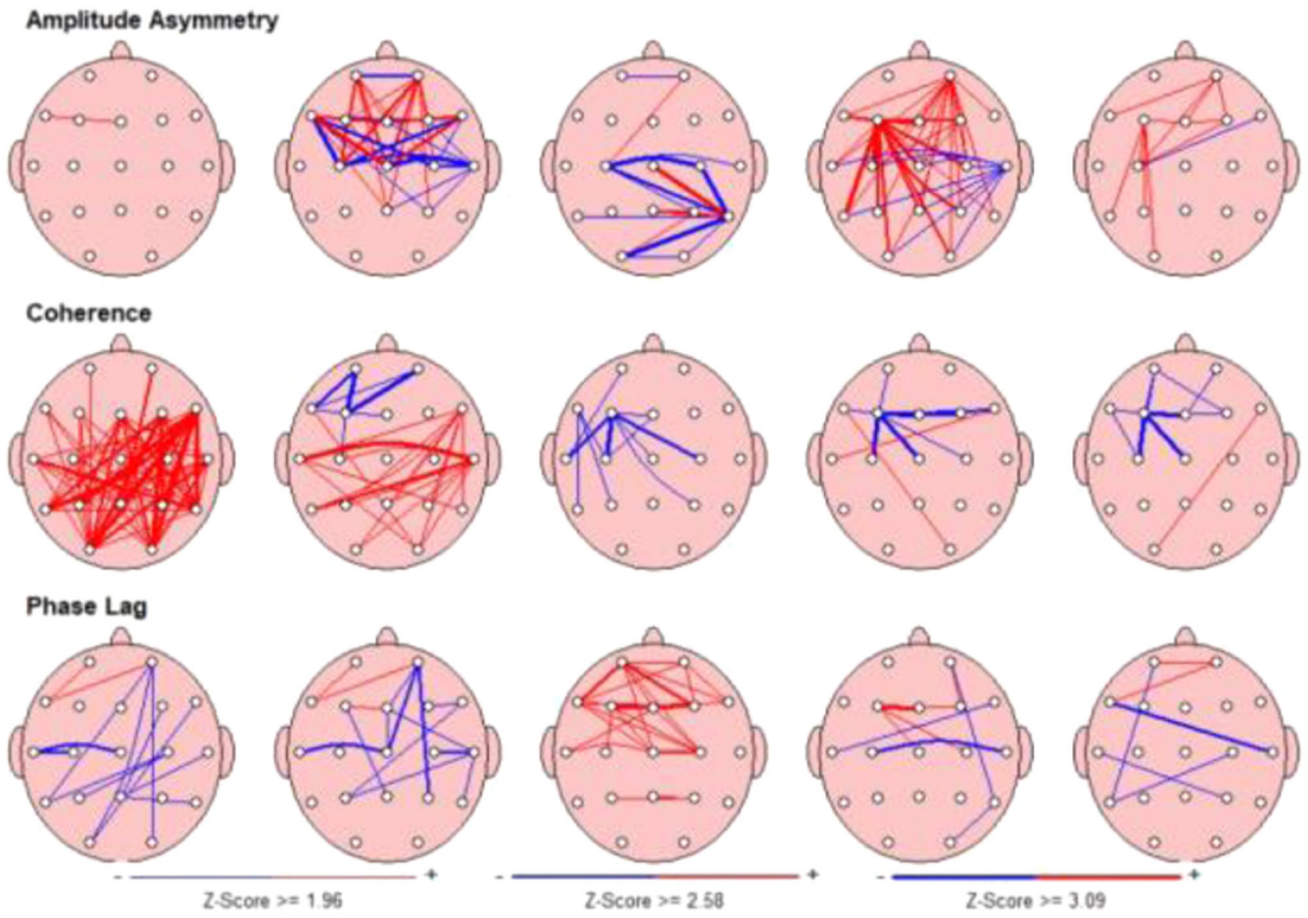
An example of QEEG summary maps in the ADHD + RD group. Hypocoherence in all three bands.

Moreover, children with ADHD with comorbid RD showed significantly lower alpha (*t* = 10.80, *p* = .045) and beta (*t* = 12.88, *p* = .009) coherence in the left frontoparietal regions. The ADHD‐alone group had lower theta (*t* = 10.90, *p* < .001) and beta (*t* = 12.36, *p* < .001) coherence in the frontal interhemispheric region compared to the comorbid group. As seen in the example of Figures 1 and 2, all differences between two groups in both frontal and parietal areas are depicted in terms of z‐scores.

## 5 DISCUSSION

The aim of the present study was to compare executive functions during resting‐state EEG by monitoring brain connectivity (coherence) patterns in children with ADHD with or without reading disability. Rs‐EEG coherence was examined across theta, alpha, and beta bands in children with ADHD with comorbid RD and compared with ADHD without RD. Consistent with previous findings in brain functional connectivity (Barry et al., 2006, 2009; Clarke et al., 2011; Robbie et al., 2016) the ADHD groups showed different brain functional connectivity profiles. Concerning our hypothesis, (1) children in the comorbid group showed reduced left frontal intrahemispheric coherence in theta, alpha, and beta bands, (2) children in the ADHD‐alone group displayed increased theta and decreased alpha and beta coherence in frontal regions, and (3) children in the comorbid group showed lower coherence between frontal and parietal networks compared to children without comorbid RD.

Our between‐group analysis of frontal regions revealed a statistically significant decreased EEG coherence in the left frontal regions in children with ADHD with comorbid RD compared to the ADHD‐alone group for theta, alpha, and beta frequency bands. As the existing body of EEG studies indicated, executive functions are associated with increased coherence in frontal theta, alpha, and beta bands, especially within medial frontal regions (Liu et al., 2019). In this regard, Ambrosini and Vallesi (2016b) demonstrated that stronger resting‐state activity in left prefrontal region is associated with better inhibition ability. Together with these findings, our current results can provide a possible hypothesis that the neural underconnectivity in frontal alpha and beta activities reflects inhibition disability in individuals with ADHD and comorbid reading disabilities.

Increased alpha coherence between frontal and parietal regions has been reported as a marker of cognitive functions particularly working memory (Babiloni et al., 2006; Sauseng et al., 2005; Shaw et al., 2015). Additionally, increases in the theta band in the left frontal and parietal regions have been associated with verbal working memory (Champod & Petrides, 2010; Olson & Berryhill, 2009). Another major finding of the present investigation was a lower EEG coherence in the alpha band in frontoparietal regions in the comorbid group compared to the ADHD‐alone group. Particularly, resting‐state functional connectivity in the left frontoparietal regions was lower than the right hemisphere. As the existing body of brain studies indicated, both ADHD and RD are associated with reduced alpha connectivity in frontal and parietal areas (Barry et al., 2009; Robbie et al., 2016). Hence, the reduced resting‐state frontoparietal coherence in the alpha band could imply the working memory dysfunction in this group. On the other hand, the other interpretation of our theta finding is therefore the verbal working memory impairment in the comorbid group.

Although not specific to the frontal region, elevated resting alpha and low beta power is thought to be positively correlated with executive function task in children (Wade et al., 2019). Others believed that these bands are known as a sign of memory retrieval and attention (Klimesch, 1999; Gruzelier, 2014). Within and between the frontal regions, both ADHD groups displayed lower alpha and beta coherences than theta. Similarly, Barry et al. (2002, 2009) reported reduced connectivity in alpha and beta in frontal regions in ADHD children. As decreased functional connectivity between and within frontal regions in high‐frequency bands is often interpreted as a disrupted cortical control circuit that likely causes impairment in cognitive task performance in children with ADHD (Hermens et al., 2005; van Dongen‐Boomsma et al., 2010), it is reasonable to say that low frontal coherence is responsible for inattention and memory impairments in individuals with ADHD.

It is believed that in cognitive tasks, the higher EEG theta power in frontal regions in children is associated with higher cognitive abilities, especially programing and control (Machinskaya & Semenva, 2004). The finding revealed that the comorbid group had lower frontal theta coherences across frontal regions. This finding is compatible with previous EEG works (e.g., Weiss & Muller, 2003) that reported reduced frontal coherence in the theta band in individuals with reading disabilities. This finding highlighted the other interpretation for our theta finding that reflects impairment in programing and control in ADHD children with comorbid reading disabilities.

On the other hands, it is well known that lower frontal theta and higher beta power in resting state are markers of attentional abilities and better executive function (especially working memory and inhibition) in children (Perone & Garstein, 2019; Perone et al., 2018). Our within‐group analysis of children with ADHD revealed significantly increased EEG coherence in theta but decreased beta coherence in frontal areas. This finding highlights the poor cognitive ability of ADHD children and supports the results from previous studies (Lansbergen et al., 2011; Robbie et al., 2016) showing that elevated theta and lower beta connectivity in frontal regions of ADHD children could be associated with impairment of inhibitory ability.

The aforementioned research findings can add to the knowledge on the association between EEG resting state coherence abnormalities and cognitive dysfunctions in children with ADHD and comorbid disabilities. In addition, the results suggest that weakness of interaction within and between frontal and posterior networks can be one factor in the executive and attentional deficits in children with ADHD and comorbid reading disabilities.

This study had several limitations. First, it had been conducted in the absence of control group. Second, executive functions were assessed in resting‐state condition. Therefore, another brain studies in addition to cognitive tasks are needed to better clarify the main reason underlying executive dysfunction in individual with ADHD and comorbid disorders. Third, the sample size for girls was small. Due to the limited access to the group of girls with ADHD, the research was conducted with a small sample size of this group.

## 6 CONCLUSION

In conclusion, the results of the present study showed that lower EEG coherence in the comorbid group and increased theta or decreased alpha and beta coherence in the ADHD‐alone group within and between frontal regions were associated with executive dysfunction in ADHD groups. Collectively, these findings converge with findings from other studies (Barry et al., 2009; Robbie et al., 2016) suggesting a relation between decreased/increased connectivity and executive dysfunction in individuals with ADHD and comorbid disabilities.

The results confirm the idea that frontal and parietal region abnormality may contribute to executive dysfunction in individuals with ADHD. We believe that EEG resting state can be useful marker for better recognize ADHD and comorbid disabilities. Therefore, by employing different cognitive tasks during EEG records we hope to better understand the relationship between EEG features and executive function in an individual with ADHD and comorbid condition.

## AUTHOR CONTRIBUTIONS

**Maryam Tabiee**: Conceptualization; data collection; methodology; writing original draft. **Ahmad Azhdarloo**: *Methodology; investigation; QEEG interpretation*. **Mohammad Azhdarloo**: *Data collection; investigation*

## CONFLICT OF INTEREST STATEMENT

The authors declare that this study was conducted in the absence of any financial support.

### PEER REVIEW

The peer review history for this article is available at https://publons.com/publon/10.1002/brb3.2951.

## Data Availability

The database used and analyzed in this study is available from the corresponding author on reasonable request.
